# Nutrient Digestibility of a Vegetarian Diet with or without the Supplementation of Feather Meal and Either Corn Meal, Fermented Rye or Rye and Its Effect on Fecal Quality in Dogs

**DOI:** 10.3390/ani11020496

**Published:** 2021-02-13

**Authors:** Amr Abd El-Wahab, Volker Wilke, Richard Grone, Christian Visscher

**Affiliations:** 1 Department of Nutrition and Nutritional Deficiency Diseases, Faculty of Veterinary Medicine, Mansoura University, Mansoura 35516, Egypt; amrwahab5@mans.edu.eg; 2 Institute for Animal Nutrition, University of Veterinary Medicine Hannover, Foundation, Bischofsholer Damm 15, D-30173 Hannover, Germany; volker.wilke@tiho-hannover.de (V.W.); sekretariat-tierernaehrung@tiho-hannover.de (R.G.)

**Keywords:** canine, dog food, nutrient utilization, fecal quality

## Abstract

**Simple Summary:**

There is a particular need to find components for more sustainability in dog foods. Rye can be produced with a very low environmental impact among cereals. Animal products like hydrolyzed feather meal cannot be used for human consumption. The aim of the present study was to evaluate the offering of a vegetarian diet supplemented with or without feather meal and either corn meal, fermented rye or rye on the fecal quality and the digestibility of nutrients in dogs. Thus, eight Beagle dogs (11.0 ± 1.31 kg) with a median age of three years were included for this study. The dogs received a vegetarian basic diet containing wheat, broken rice and rice protein or the same diet supplemented with hydrolyzed feather meal (2.7%) and either 20.1% of corn meal, 60.4% of fermented rye or 20.1% of rye as is basis (moisture content of the diets about 42%). During the study, the feces were well formed and firm and the digestibility of the diet was not different by using rye compared to all other diets. Moreover, the acceptance “food intake scoring“ was similar among the experimental diets fed to dogs. Therefore, rye can be considered as an alternative cereal grain in dog foods.

**Abstract:**

Cereals with low environmental input like rye and animal by-products which cannot be used for human food like feather meal are receiving growing interest as sustainable feed sources. Thus, eight Beagle dogs were included in a 4 × 4 Latin Square design and received a vegetarian basic diet or the same diets supplemented with hydrolyzed feather meal (2.7%) and either 20.1% of corn meal, 60.4% of fermented rye or 20.1% of rye as is basis (moisture content of the diets about 42%). Compared to other groups the dry matter (DM) content of feces from dogs fed the basic diet was higher (30.0%, *p* < 0.05), while dogs fed the basic diet + rye had the lowest DM-content (26.5%, *p* < 0.05). However, the fecal scores were considered to be within an acceptable range (well-formed and firm). Starch digestibility was lower (*p* < 0.05) for dogs fed the basic diet + corn meal. The dogs showed a high and identical acceptance (scoring of food intake) of the experimental diets. As a comparable quality of feces and a high nutrient digestibility were observed when rye was used in the experimental diets—it can be considered an alternative carbohydrate source in dog foods.

## 1. Introduction

Several factors can impact the nutritional sustainability of a food system, including the ingredient selection, nutrient composition, food intake and digestibility [1]. Thus, it means to have more sustainable grain to be fed for animals, its nutritive value as well as its palatability is important. Cereal grains usually represent 30–60% of the dry matter in dry foods for dogs and provide energy [2]. Rye is more tolerant to water shortages than wheat, however it is still not commonly used in dog diets [3]. Additionally, rye is characterized by a low carbon footprint and moreover offers significant cost savings compared to wheat [4]. In terms of sustainability, animal products like feather meal, which otherwise would not find hardly any use, could be included in the diet in certain levels without negative impacts on animals’ health [5]. In the last decade, the global prevalence of those people who have lessened their intake of or avoid eating animal products completely has increased [6]. Health concerns and sustainability/environmental preservation are considered the main motivations for this shifting away from animal products [7,8]. Pet owners, who avoid eating animals often feed or are interested in feeding plant-based or vegan diets to their pets and report feelings of guilt and internal conflict regarding feeding animal products to their pets [9,10]. However, in terms of sustainability, it would be preferable to use rye as a cereal with a low negative impact on the environment and, moreover small amounts of animal products like feather meal that would otherwise remain unused. This would probably meet the owners’ demands for a more sustainable diet rather than a strictly vegetarian diet. Besides the parameter of sustainability, rye is experiencing growing interest of research in animal nutrition especially because of the unique carbohydrate fraction such as arabinoxylans and fructans [11]. Due to its special composition, it inevitably affects various processes in the digestive tract [11,12]. Besides this, the digestion of previously undigested components (dietary fibers) takes place microbially with the formation of short-chain fatty acids in the large intestine [13]. High levels of short-chain fatty acids lead to increased intestinal energy supply and enhanced growth of beneficial microorganisms [14]. Furthermore, it can be presumed that an accumulation of short-chain fatty acids and especially butyric acid in the intestine due to a diet containing higher levels of resistant starch or dietary fiber also has positive effects on the supply and health of the intestinal wall in dogs [15,16,17]. Fiber type (soluble vs. insoluble fibers) and the concentration of the fiber in the diet can impact nutrient utilization and stool quality [18]. Moreover, dog foods contain fiber may promote and regulate normal intestine function by increasing bulk and water content of the chyme and alteration of the intestinal transit [19]. Thus, the high amounts of dietary fiber might have an impact on parameters of digestibility and fecal characteristics when feeding rye to dogs. However, studies on including rye (ll fermented or not) in dog foods are not yet available. Due to limited published information, the aim of the present study was to determine the effects of a vegetarian diets supplemented with or without hydrolyzed feather meal and either corn meal, fermented rye or rye on fecal quality and the nutrient digestibility of dogs in comparison to wheat and rice.

## 2. Materials and Methods

The study design was approved by the Animal Welfare Officer of the University of Veterinary Medicine Hannover, Foundation, Germany.

### 2.1. Study Design

Eight healthy female Beagle dogs (*n* = 8) were included in the digestibility study at the Institute for Animal Nutrition, University of Veterinary Medicine Hannover, Foundation, Germany. At the beginning of the study the dogs had a mean body weight (BW) of 11.0 ± 1.31 kg with a median age of three years (range 28–76 months). The dogs lived in 3.35 × 2.80 m kennels with daily access to an outside playground for exercise and socialization, where they were acclimatized to the experimental diets. During the digestibility tests, dogs were housed individually in 4.00 × 2.05 m special kennels to enable fecal collection and at the same time to enable social contact between the animals. During this period, the dogs had free access to water anytime.

The dogs received the different diets according to a 4 × 4 Latin Square experimental design. During this study, each dog was assigned once to a vegetarian basic diet or the same diet supplemented with hydrolyzed feather meal and either corn meal, fermented rye or rye, respectively. The diet meal (about 295 g/dog/d, except in case of feeding basic diet + fermented rye + feather meal it was about 289 g/dog/d) was offered once a day for all dogs at the same time according to their energy requirements. In the digestibility trial, each period lasted a total of ten days including a five-day adaptation period [20,21,22]. Four days were used as wash-out before each experimental period (adaptation + collection). The total amount of feces and apparent nutrient digestibility were determined individually for each animal. The amount of fresh water was weighed and offered every day in a metal container. After each day of the trial the residual amount of water was weighed to calculate the daily water intake.

### 2.2. Diet Preparation

An extruded basic diet (MERA Tiernahrung GmbH, Kevelaer, Germany) was produced as a vegetarian diet and composed of wheat, broken rice, wheat gluten, rice protein, linseed, sunflower oil and dried beet pulp (Table 1). The other three non-vegetarian diets were obtained by adding hydrolyzed feather meal to the basic diet (2.7% as is basis). These non-vegetarian diets were further supplemented by either corn meal (basic diet + corn meal), fermented rye (basic diet + fermented rye) or rye (basic diet + rye). The corn and rye (variety: KWS Trebiano) grains were provided by KWS LOCHOW GmbH, Bergen, Germany. These grains were grinded to be about 1 mm in diameter. All of the feed ingredients as hydrolyzed feather meal, corn meal, fermented rye and rye were added and manually mixed to each dietary meal daily. Additionally, the supplemented corn meal and rye were not cooked before the addition to the basic diet as possible effects of fermentation could be more clearly described. Moreover, 120 mL of warm water was added at each feeding meal to all the diets (except basic diet + fermented rye) to achieve similar consistency among diets. The fermented rye was quite moist (about 307 g/kg dry matter (DM)), so there was no need to add water to the basic diet + fermented rye.

The fermented rye was produced by mini-fermenters (Mini-Fermenter 125 L, WEDA Dammann & Westerkamp GmbH, Goldenstedt, Germany). Briefly, after filling the mini-fermenter (rye to water ratio of 1:3), it was closed and the liquid feed was stirred therein for 60 s at 900 rpm. The fermentation temperature of 35–38 °C was achieved for the entire fermentation period (24 h). To avoid malfermentation, a freeze-dried, granulated starter culture (Schaumalac Feed Protect XP G, H. Wilhelm Schaumann, Germany), consisting of 1k2079 *Lactobacillus plantarum*, 1k2103 *Pediococcus pentosaceus* and 1k2082 *Lactococcus lactis*, was added at the beginning of each fermentation process at a dosage of 2 × 10^5^ CFU/g ingredient.

### 2.3. Diet Analyses

In accordance with VDLUFA (Verband Deutscher Landwirtschaftlicher Untersuchungs- und Forschungsanstalten) methods [23], all ingredients, as well as the formulated diets were analyzed for DM, crude ash, crude fat, crude protein and crude fiber before formulation (Table 2 and Table 3).

The DM content was determined by drying at 103 °C to a constant weight, while the muffle furnace was used to determine the content of crude ash for 6 h at 600 °C. The crude fat content was determined in the Soxhlet apparatus. The total nitrogen content was determined by elemental analyzer (Elementar Analysensysteme GmbH, Hanau, Germany), which operates in accordance with the DUMAS combustion method, while the content of crude fiber was determined after washing in dilute acids and alkalis. Starch determination was measured polarimetrically (Polatronic E, Schmidt und Haensch GmbH & Co., Berlin, Germany). The sugar content was analyzed in accordance with the principals of Luff-Schoorl method by titration with sodium thiosulfate [23]. The mineral content was carried out by atomic absorption spectrometry (Unicam Solaar 116, Thermo Fisher Scientific GmbH, Dreieich, Germany) in accordance with AOAC [24]. Contents of lysine, methionine, threonine and tryptophan in the basic diet were calculated by BESTMIX^®^ (adifo software, Maldegem, Belgium) based on values determined by internal analyses of the animal feed producer. For calculating lysine, methionine, threonine and tryptophan contents in the non-vegetarian diets, values for rye and corn meal were taken from Rodehutscord et al. [11] and Schulten [25], while for feather meal these were taken from Adejumo et al. [26]. In the case of the fermented rye, the same values were taken for the calculation as for rye because previous analyses have shown that amino acid contents do not differ after the fermentation process [27]. The amount of diet provided was individually determined using standard equations for the daily energy requirements of kennel dogs (0.45 MJ metabolizable energy /kg body weight ^0.75^/d). Metabolizable energy (ME) contents of the diets were estimated based on their chemical compositions, in accordance with the NRC [28]. In accordance with Zahn [29] the spontaneous acceptance “food intake scoring“ (palatability and the speed of food intake) was divided into 3 grades (1 = lowest acceptance; 2 = middle acceptance; 3 = highest acceptance).

### 2.4. Apparent Digestibility

**Figure 1:**
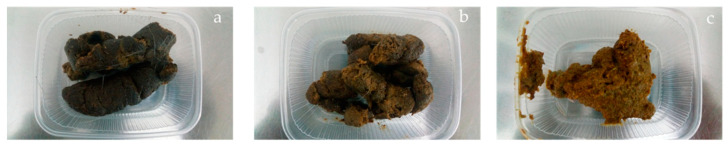
Fecal scores in accordance with Moxham [31]: (**a**) Score 2 (solid, well formed); (**b**) Score 3 (soft, still formed); (**c**) Score 4 (pasty, slushy); Foto: Abd El-Wahab, A. ^©^TiHo.

During five consecutive days (last five days of the ten days period), the feces were collected completely and individually every two hours. At the end of each day of collection phase, the collected feces were thawed, mixed and homogenized to receive an individual daily fecal sample. In a sample of 10% of the fresh feces per animal and day, DM content was determined on each day of the collection phase. The remaining 90% of the sample was frozen at −20 °C. By mixing the daily fecal pool samples of an animal into one total pool sample at the end of each collection phase, it was finally possible to create a collective subsample of each animal. The apparent digestibility was calculated according to the following formula: Apparent digestibility (%) = ((food-excreta)/food) × 100 [30]. The number of defecations was recorded every day. In accordance with Moxham [31] during collection, fecal scores (Figure 1) were recorded using a 5-point scale (1 = very hard; 2 = solid, well formed “optimum”; 3 = soft, still formed; 4 = pasty, slushy and 5 = watery diarrhea).

### 2.5. Statistical Analyses

The statistical analysis was performed using the Statistical Analysis System for Windows, SAS^®^ Enterprise Guide^®^, version 9.3 (SAS Institute Inc., Cary, NC, USA). For all parameters, mean values as well as the standard deviation of the mean were calculated. All measured or recorded parameters were analyzed individually and were the basis of the calculation. A test for normal distribution was performed by means of distribution analysis using the Shapiro-Wilk test for analytical evaluation. Depending on this, parametric and non-parametric methods were used. Normally distributed model residuals were tested by analysis of variance or by a multi-range test (Ryan Einot-Gabriel-Welsch test). For non-normally distributed data or values in the form of a score, the Kruskal-Wallis test with post-hoc tests in accordance with Dwass, Steel and Critchlow-Flinger for multiple two-sided paired comparisons was applied accordingly. The significance level was determined at *p* < 0.05.

## 3. Results

### 3.1. Dietary Treatments

The analyzed chemical composition of the raw ingredients is shown in Table 2. The crude protein content for corn meal, fermented rye and rye is almost identical (111–113 g/kg DM), while crude protein content for feather meal is about 884 g/kg DM. Marked differences were found in the sugar content between the different raw ingredients (0.90–80.1 g/kg DM). The analyzed chemical composition of the experimental foods fed to dogs is found in Table 3. The moisture content among diets was nearly similar with a slight decrease in the basic diet + corn meal (about 397 g/kg). Slight deviations in crude protein and crude fat were noted. The crude protein level was slightly higher for the basic diet (220 g/kg DM) than the other diets (mean: 215 g/kg DM). The crude fat content was highest in the basic diet + corn meal and basic diet + fermented rye (72.7 and 71.2 g/kg DM, respectively) relative to the other diets. Minor differences in fiber, starch and sugar contents occurred due to the individual cereal composition. Regarding the crude fiber content, the basic diet + corn meal was 4.60 g/kg DM greater than the basic diet + rye. The starch was higher in the basic diet + rye and basic diet + fermented rye (average 482 g/kg DM) and markedly lower in the basic diet + corn meal (435 g/kg DM). A marked decrease in the sugar concentration in the basic diet (26.3 g/kg DM) was noted in comparison to the other diets (mean 43.4 g/kg DM).

### 3.2. Food Intake and Body Weight

All dogs readily ate all diets and no signs of dietary refusal were observed (Table 4). Both the proportion of rye and of fermented rye were accepted without problems; food refusals were not noted. The spontaneous food intake scoring was identically high (degree 3) for all dogs between all dietary treatments. Water intake showed differences (*p* < 0.05) between the experimental groups (Table 4). Dogs fed either the basic diet + corn meal or basic diet + fermented rye had higher (*p* < 0.05) water intake (489 and 498 g, respectively) compared to dogs fed the basic diet or basic diet + rye (344 and 380 g, respectively). Dogs fed the basic diet + fermented rye displayed the highest (*p* < 0.05) water:food intake ratio (2.95) compared to those fed the basic diet (2.11). However, dogs fed either the basic diet + corn meal or basic diet + rye showed no significant differences in the water:food intake ratio (2.75 and 2.25, respectively). Food allowance was intended to maintain animal body weight. Thus, a weight loss was not observed in this study.

### 3.3. Fecal Quality

In the present study, the defecation frequency (average 2.11) was not significantly affected by the type of the ingredient added to the diet (Table 5). Additionally, the total amount of collected feces did not differ significantly for dogs fed experimental diets. The DM content of feces analyzed is presented in Table 5. Compared to other groups the DM content of feces from dogs fed the basic diet had the highest (*p* < 0.05) values (30.0%), while dogs fed the basic diet + rye diets exhibited the lowest DM content of feces (*p* < 0.05). Generally, fecal scores were considered acceptable, with an average score of 2.79. Furthermore, dogs fed the basic diet + rye and basic diet + corn meal diets displayed high (*p* < 0.05) fecal scores in comparison to other groups, while dogs fed the basic diet showed the lowest fecal score.

### 3.4. Apparent Digestibility

The apparent digestibility in dogs fed the experimental diets is shown in Table 6. No significant differences were observed for apparent digestibilities of organic matter, crude protein and crude fat (range: 84.1–85.8%, 76.4–80.0% and 86.5–89.1%, respectively). Starch digestibility was lower (*p* < 0.05) for dogs fed the basic diet + corn meal (96.1%), whereas no significant differences were observed among dogs fed the basic diet, basic diet + rye or basic diet + fermented rye diets.

## 4. Discussion

Several factors may affect the digestibility of a diet, including the ingredient source and its chemical composition [32]. The mean DM content for all the experimental diets was about 57.9% ± 22.1. To ensure comparable DM contents among the diets, especially with the diet containing fermented rye, all diets were offered in a semi-moist state. Also, the concentrations of nutrients, especially the content of crude protein (217 g/kg DM ± 3.01), were similar among the diets.

Palatability is the measure of intake of a food that indicates acceptance or the measure of preference of one food over another [33]. As shown in previous investigations [34], the dogs in this study also showed a high acceptance for the semi-moist diets. Nevertheless, [35] it was observed that increasing dietary moisture for dogs did not affect food selection. The reasons for the higher water intake for groups fed the basic diet + corn meal and basic diet + fermented rye diets are unclear. It is known, that adding fiber-rich components in dog foods increase water intake depending on the type of fiber [36]. The highest fiber-content was measured in the diet in which corn was added and with this the highest water intake occurred. However, the absolute differences in fiber content were not high enough to be relevant for an explanation. Compared to basic diet, the DM-contents of feces were lower in dogs fed the experimental diets, which could explain in part the generally higher water intake by dogs in these groups due to higher water losses by feces. Additionally, the corn meal, fermented rye and rye were provided uncooked and, consequently, it could lead to more fermentation in the colon of these particular diets and that might be the main cause of the increase in fecal moisture. Moreover, Mühlum [37] stated that the moisture content in feces of dogs increased when feeding carbohydrates with high postileal digestibility (lactose, starch). It means that there was a close relation between fecal excretion of lactic acid and the amount of fecal moisture.

In our study, the frequency of defecations among the groups were not different, however, the crude ash contents among diets differed. In a study by Zeiger [38], a high defecation frequency (3.2 per day) was observed when dogs were fed a diet with a high crude ash content (141 g/kg DM). However, it was not the case in our study as it was found that the dietary crude ash contents (range: 41.3–53.8 g/kg DM) had no effect on the defecation frequency. It could be that the dietary crude ash contents in our study were not high enough to exert an effect on the frequency of defecation. Fecal consistency can be determined by either fecal consistency scores or fecal DM content [39]. Moreover, the fecal quality is a major factor by which dog owners judge a commercial food [39]. In the present study, the fecal scoring was different among dogs fed the different diets, however, it was generally about 2.79 ± 0.32 (close to the optimal score value of 2). Ingenpaß [21] observed no statistical correlation between the DM content and the visual consistency (score) of the feces. Sunvold et al. [39] concluded that the fecal consistency score was more indicative of fecal characteristics than DM content. However, according to Meyer and Zentek [40], the fecal consistency is mainly determined by the moisture content in the feces. Because fecal quality and score are very essential to pet owners, any change in fecal consistency could be seen as unfavorable to the use of an ingredient. Nevertheless, Fricke [41] stated that undigested proteins act as water-binding factors in the chyme and consequently affect fecal moisture content (R^2^ = 0.25). However, it was a low correlation, so likely fecal protein has little impact on fecal moisture. Thus, it cannot be ruled out that a part of feather meal in the diet ended up in the large intestine and was fermented to some extent. Unlike starch in the diet, fiber is not digested by the animal’s digestive enzymes. Feeding dogs a moderate fermentable fiber source led to a part of the fiber being fermented and utilized by the microorganisms in the colon, consequently less organic material is excreted in the feces [42]. According to Zentek [43] the different water binding capacities of fiber sources as well as the quality of the plant fiber per se (soluble or insoluble) are of particular interest. This increased fermentative activity in large dogs could contribute to the poorer quality of their stool. High positive correlations were found in dogs between fermentation activity on the one hand and water content or consistency of stool on the other [36]. However, in our study we did not measure the insoluble fiber and soluble fiber contents of the diets. According to Rodehutscord et al. [11] the contents of neutral detergent fiber, acid detergent fiber and acid detergent lignin were 88.9, 27.4, 4.50 g/kg DM for corn, respectively and 146, 29.6, 8.58 g/kg DM for rye, respectively. Thus, in our study, it is expected that the contents of neutral detergent fiber and acid detergent lignin will be higher in the basic diet supplemented with rye compared to the basic diet supplemented with corn meal. According to Siebert [44], with higher amount of crude fiber in the compound food the fecal mass increased.

The apparent digestibility of organic matter between all experimental diets were not different. There is a generally belief that increased fiber results in a reduction of apparent nutrient digestibility [20]. In our study, the apparent organic matter digestibility was comparable among the dietary treatments. Additionally, the crude fiber in the diets was considered low (range: 17.0–21.6 g/kg DM) in the present study compared to Schulten [25], who found that the crude fiber content in commercial diets were about 28.2–32.5 g/kg DM. Thus, it could be that the low dietary crude fiber content in our study was not high enough to have an effect on the apparent digestibility of organic matter. The European Pet Food Industry Federation [45] states that an apparent protein digestibility of 80% was used to establish the protein minimum recommendation, which approximately corresponds to our results. Although the biological value of feather meal is low, compared to the other added components, the amounts of essential amino acids were markedly higher [46]. The nutritional composition of feather meal can differ widely due to the processing type [47]. Currently, the most economical method for hydrolysis of feathers is by using high temperature and pressure, resulting in an ingredient with high levels of protein but poor amino acid bioavailability [48]. Nevertheless by adding only small amounts (2.7%) of the hydrolyzed feather meal to the diet it might not influence the chemical composition of the diets particularity. Schulten [25] used hydrolyzed feather meal at a concentration of 25% and determined crude protein digestibility in dogs amounting to about 78.2%. In other studies by Siebert [44] and Zeiger [38], feeding dogs with about 33.3% and 20% hydrolyzed feather meal, resulted in crude protein digestibility of about 75.9% and 81.1%, respectively. In principle, it can be assumed that very high proportions of feather meal in food do not lead to a higher digestibility, compared with more moderate proportions [49]. However, the small amounts of feather meal, used in our study, might not have had a particularly high impact on parameters of digestibility. Besides the general influence of the raw materials used and the processing, there are other factors influencing the digestibility of a diet. Several antinutritional factors in ingredients of vegetable origin could influence the digestibility [50,51]. Among others, in particular the high amounts of non-starch-polysaccharides of rye have an impact, particularly on the prececal digestibility in monogastric species [52]. Moreover, it is well known that protein of plant origin generally have a lower digestibility than protein of animal origin [53]. Nevertheless, in the present study, the apparent protein digestibility was comparable among all the experimental diets.

## 5. Conclusions

The inclusion of rye or fermented rye in dog diets did not affect the spontaneous intake of food. Dogs fed the vegetarian diet (basic diet) and the same diet supplemented either with corn meal, fermented rye or rye generally achieved a good fecal score regardless of the fecal dry matter content. Despite the differences in diet composition, the digestibility of organic matter, crude protein, crude fat and N-free extract were not affected for any of the diets. Further studies are needed to investigate the possibilities of using rye in higher dietary levels for dogs when a higher acreage of rye is intended for sustainability reasons.

## Figures and Tables

**Table 1:** Components of the vegetarian diet (basic diet) and the experimental diets supplemented with feather meal as well as either corn meal, fermented rye or rye.

Ingredient (% as is Basis)	Basic Diet	Basic Diet + Corn Meal	Basic Diet + Fermented Rye	Basic Diet + Rye
Wheat	17.3	10.7	10.7	10.7
Broken rice	17.3	10.7	10.7	10.7
Wheat gluten	5.2	3.3	3.3	3.3
Rice protein	5.2	3.3	3.3	3.3
Sunflower oil	4.1	2.5	2.5	2.5
Dried beet pulp	1.8	1.1	1.1	1.1
Brewer’s yeast	1.2	0.7	0.7	0.7
Linseed	1.2	0.7	0.7	0.7
Lignocellulose	0.5	0.3	0.3	0.3
Sum of additives ^1^	5.7	3.5	3.5	3.5
Feather meal	-	2.7	2.7	2.7
Corn meal	-	20.1	-	-
Rye	-	-	-	20.1
Fermented rye ^2^	-	-	60.4	-
Water	40.5	40.3	-	40.3

^1^ Dicalcium phosphate, sodium chloride, potassium chloride, choline chloride, _L_-lysine, _DL_-methionine, magnesium carbonate, taurine, protein hydrolysates with additives such as synthetic amino acids, sugars and phosphoric acids and antioxidants. ^2^ Dry matter content: 30.7%. Sum of percentages may deviate from 100% due to rounding up.

**Table 2:** Chemical composition (in g/kg DM) of hydrolyzed feather meal, corn meal, fermented rye and rye used in dog foods.

Parameter	Feather Meal	Corn Meal	Fermented Rye	Rye
Dry matter in fresh matter	901	902	307	869
Crude ash	31.0	21.9	19.6	17.2
Crude fat	88.6	40.8	19.7	19.7
Crude protein	884	113	111	113
Crude fiber	5.70	26.0	20.0	20.0
Starch	0.00	662	585	608
Sugar	0.90	27.9	80.1	67.4
Calcium	4.96	1.80	1.80	0.55
Phosphorus	3.77	3.80	3.30	3.10
Lysine	26.6	2.98	3.59	3.59
Methionine	8.40	2.06	1.52	1.52
Threonine	37.7	3.65	3.23	3.23
Tryptophan	5.60	0.75	1.02	1.02

**Table 3:** Chemical composition (in g/kg DM) of the vegetarian diet (basic) and the experimental diets supplemented with hydrolyzed feather meal as well as either corn meal, fermented rye or rye.

Parameter	Basic Diet	Basic Diet + Corn Meal	Basic Diet + Fermented Rye	Basic Diet + Rye
Dry matter in fresh matter	551	603	587	573
Crude ash	53.8	42.6	41.8	41.3
Crude fat	67.5	72.7	71.2	62.9
Crude protein	220	218	214	214
Crude fiber	18.3	21.6	18.0	17.0
N-free extract	640	645	655	665
Starch	468	435	483	482
Sugar	26.3	42.2	39.9	48.0
ME (MJ/100 g as fed) ^1^	0.89	0.98	0.96	0.93
Calcium	9.92	6.85	7.00	6.86
Phosphorus	3.86	3.01	3.09	2.99
Lysine	10.0	7.41	8.02	8.22
Methionine	6.13	4.29	4.35	4.45
Threonine	6.33	6.34	6.60	6.76
Tryptophan	2.25	1.74	1.93	1.98

Calculated values of total dietary fiber for the four diets (except for wheat gluten and rice protein) were 113, 135, 122 and 122 g/kg dry matter, respectively. ^1^ Metabolizable energy (ME) content of the diets was estimated in accordance with the NRC [28]. Amino acid contents were calculated. Sums of crude ash, crude fat, crude protein, crude fiber, N-free extracts may not total 1000 g due to rounding up.

**Table 4:** Food intake on DM basis, food intake scoring, water intake and BW of dogs fed vegetarian diet (basic) and the experimental diets supplemented with hydrolyzed feather meal as well as either corn meal, fermented rye or rye (mean ± SD).

Item	Basic Diet	Basic Diet + Corn Meal	Basic Diet + Fermented Rye	Basic Diet + Rye	*p*-Value
Food intake (g)	162 ^c^ ± 0.901	178 ^a^ ± 1.65	170 ^b^ ± 2.54	169 ^b^ ± 1.73	<0.0001
Food intake scoring (1–3)	3.00 ± 0.00	3.00 ± 0.00	3.00 ± 0.00	3.00 ± 0.00	1.000
Fresh water intake (g)	344 ^b^ ± 112.81	489 ^a^ ± 159.31	498 ^a^ ± 275	380 ^b^ ± 226	0.0008
Food water intake (g)	132 ^a^ ± 0.735	117 ^c^ ± 1.09	119 ^c^ ± 1.79	126 ^b^ ± 1.29	<0.0001
Water:food intake ratio	2.11 ^b^ ± 0.698	2.75 ^ab^ ± 0.893	2.95 ^a^ ± 1.64	2.25 ^ab^ ± 1.35	0.0006
BW at start (kg)	11.0 ± 1.31	11.1 ± 1.34	10.9 ± 1.29	11.0 ± 1.26	0.9929
BW at final (kg)	10.8 ± 1.29	11.0 ± 1.35	11.0 ± 1.29	10.8 ± 1.24	0.9897

^a,b,c^ Means in a row with different superscripts differ significantly (*p* < 0.05). Water intake from the diet (g) is about 133; 117; 119 and 126 for basic diet, basic diet + corn meal, basic diet + fermented rye and basic diet + rye, respectively.

**Table 5:** Number of defecations, average daily amount of feces, fecal score, and amount of feces g DM/100 g ingested diet DM of dogs fed the vegetarian diet (basic) and the experimental diets supplemented with hydrolyzed feather meal as well as either corn meal, fermented rye or rye (mean ± SD).

Item	Basic Diet	Basic Diet + Corn Meal	Basic Diet + Fermented Rye	Basic Diet + Rye	*p*-Value
Defecation (n/d)	2.29 ± 0.825	2.09 ± 0.781	1.86 ± 0.733	2.20 ± 0.719	0.1087
Average amount of feces (g/d)	95.5 ± 30.5	112 ± 51.4	97.6 ± 36.1	115 ± 34.0	0.0640
Fecal score (1–5)	2.39 ^c^ ± 0.575	3.09 ^a^ ± 0.502	2.68 ^b^ ± 0.522	3.00^a^ ± 0.550	<0.0001
Fecal DM (%)	30.0 ^a^ ± 1.96	27.9 ^b^ ± 2.52	27.6 ^b^ ± 1.53	26.5^c^ ± 1.37	<0.0001
Amount of feces (g DM/100 g ingested diet DM)	17.6 ± 5.48	17.5 ± 8.09	15.8 ± 5.92	17.9 ± 5.42	0.3044

^a,b,c^ Means in a row with different superscripts differ significantly (*p* < 0.05).

**Table 6:** Apparent nutrient digestibility (%) of dogs fed the vegetarian diet (basic) and the experimental diets supplemented with hydrolyzed feather meal as well as either corn meal, fermented rye or rye (mean ± SD).

Item	Basic Diet	Basic Diet + Corn Meal	Basic Diet + Fermented Rye	Basic Diet + Rye	*p*-Value
Organic matter	85.0 ± 1.09	84.1 ± 2.74	85.8 ± 2.03	83.5 ± 2.34	0.2629
Crude protein	79.9 ± 1.66	79.1 ± 3.50	80.0 ± 2.91	76.4 ± 4.25	0.2124
Crude fat	88.8 ± 1.03	88.0 ± 1.91	89.1 ± 1.67	86.5 ± 2.47	0.0679
Starch	98.9 ^a^ ± 0.357	96.1 ^b^ ± 1.95	98.9 ^a^ ± 0.354	97.9 ^a^ ± 0.587	<0.0001
Sugar	84.3 ± 2.92	89.9 ± 2.66	88.7 ± 5.04	86.1 ± 4.49	0.0499
N-free extract	89.0 ± 1.11	87.5 ± 2.58	89.4 ± 1.74	87.7 ± 1.74	0.1916

^a,b^ Means in a row with different superscripts differ significantly (*p* < 0.05).

## Data Availability

The original contributions generated for the study are included in the article, further inquiries can be directed to the corresponding author.
